# Development of the German social attitude barriers and facilitators to participation-scales: an analysis according to the Rasch model

**DOI:** 10.1186/s12891-022-05339-0

**Published:** 2022-05-06

**Authors:** Luz Dary Upegui-Arango, Verena Mainz, Judith Gecht, Christian-Andreas Mueller, Valentin Quack, Allen W. Heinemann, Maren Boecker

**Affiliations:** 1 Institute of Medical Psychology and Medical Sociology, University Hospital of RWTH Aachen University, Aachen, Germany; 2 Clinic for Anesthesiology, University Hospital RWTH Aachen, Medical Faculty, RWTH Aachen, Aachen, Germany; 3 AIXTRA – Competence Center for Training and Patient Safety, Medical Faculty, RWTH Aachen, Aachen, Germany; 4 Department of Neurosurgery, University Hospital of RWTH Aachen University, Aachen, Germany; 5 Department of Orthopaedic Trauma and Reconstructive Surgery, RWTH Aachen University Hospital, Aachen, Germany; 6 Department of Physical Medicine and Rehabilitation, Feinberg School of Medicine, Northwestern University, Chicago, IL USA; 7 Center for Rehabilitation Outcomes Research, Shirley Ryan AbilityLab, Chicago, IL USA

**Keywords:** Social attitudes, Disabilities, Environment, Patient-reported outcomes, Rasch analysis, Item response theory

## Abstract

**Background:**

Social attitudes experienced by people with disabilities can strongly impact upon their health and quality of life. The extent to which social attitude measurement transcends specific cultures is unknown. Thus, the aim of the study was to develop German item banks to assess social attitude barriers and facilitators to participation and compare the construct definition with that developed in the United States.

**Methods:**

The American version of the two item banks assessing social attitudes that act as barriers and facilitators in persons with disabilities was translated into German and culturally adapted. The sample consisted of 410 in- and outpatients treated for spinal diseases at a German University Hospital. The psychometric properties of the resulting 53 items-item pool were evaluated using Rasch analysis. A special focus was placed on the investigation of unidimensionality, local independence, differential item functioning (DIF) and targeting. To evaluate convergent and divergent validity correlations with perceived social support, depression and pain interference were calculated.

**Results:**

Unlike the American version, both the barriers and facilitators item banks had to be divided into two subscales assessing attitudes that individuals with disabilities experience as being directed towards them (individual perception) or attitudes that respondents experience as being directed towards people with disabilities as a social group (societal perception). Four unidimensional scales were constructed. Fit to the Rasch model required item deletion and forming testlets to account for extensive local dependence. There was no evidence of DIF with regard to gender or age. Targeting of the subscales was moderate to good.

**Conclusions:**

Results support a distinction between social attitudes at the individual and societal level, allowing a more specific assessment than is possible when this distinction is ignored.

**Supplementary Information:**

The online version contains supplementary material available at 10.1186/s12891-022-05339-0.

## Introduction

Worldwide, the number of people with disabilities is increasing and with it the individual and social costs. In relation to this, the Word Health Organization (WHO) developed the International Classification of Functioning, Disability and Health (ICF), in which disability is viewed as a result of a dynamic interaction between health conditions and personal, contextual and environmental factors [1]. Environmental factors such as social attitudes can promote or restrict the participation of persons with disabilities in society and can have a profound impact upon their quality of life. In this sense, the attitudes of family, friends, and society regarding people with disabilities, can be a strong barrier or facilitator to participation.

Attitudinal barriers such as stereotyping, stigma, prejudice, and discrimination can contribute to other barriers that range from physical to systemic, since attitudinal barriers may limit recognition and resolution of the difficulties experienced by people living with disabilities. In contrast, attitudinal facilitators such as inclusion, acceptance, integration, respect and social equity, help to ensure their participation in society [2]. In doing so, the impact of attitudinal barriers and/or the absence of attitudinal facilitators may vary from limited social support, limited participation and low quality of life to long-term health impacts [3].

Therefore, it is important to have psychometrically solid assessment tools for assessing social attitudes experienced by people with disabilities as a group on a societal level as well as those experienced by the individual in the personal environment. To meet the need for a holistic measurement of social attitudes, Garcia and colleagues developed an item pool of 82 items based on a social environment conceptual framework [4]. They explicitly considered social attitude facilitators and barriers and included items reflecting either lived experiences of people with disabilities as a group in general or the experiences of individuals in their personal environments. Wong and colleagues [5] evaluated the psychometric properties of these items in a mixed patient group (stroke, spinal cord injury, traumatic brain injury) using the Rasch model. They developed two item banks – one for the social attitude facilitators and one for the social attitude barriers. Both item banks showed a good spread of item difficulties and good reliability (.99 for both item banks). For some items, differential item functioning (DIF) was observed, but as the authors appraised the size of DIF as minimal, they retained these items in the final item banks.

The present study had the objective to evaluate the construct validity of the social facilitators and barriers item banks by adapting culturally and calibrating a German version of the item banks developed by Garcia and colleagues [4], and Wong and colleagues [5]. For the German version, 53 of the 82 items were selected, translated and evaluated in a sample of patients with spinal diseases (SpD). Like in the study of Wong and colleagues [5] the psychometric properties of the items were evaluated by means of the Rasch model. Besides unidimensionality, DIF, targeting, item fit as well as overall fit to the model, we investigated local dependence (LD) across items, an issue not reported in the previous studies. However, since locally dependent items can lead to the estimation of biased parameters and to the inflation of reliability [6–8], it is crucial to evaluate and ameliorate it.

Convergent validity was assessed, to investigate the possible association between the social attitude facilitators and barriers with perceived social support as assessed with the Perceived Social Support Questionnaire (F-SozU-22) [9]. Additionally, the associations between the evaluated self-report social attitudes measures, the Rasch-based depression screening (DESC-I) [10, 11] and the Pain Interference Scale-German (PI-G) [12] were evaluated.

## Methods

### Participants and data collection

The study was part of a larger project with in- and outpatients treated for SpD in Germany [13]. The sample consisted of 410 patients with a diagnosis of SpD according to the 10th revision of the International Classification of Diseases (ICD-10) [14]. Exclusion criteria were age under 18 years, severe cognitive impairment, insufficient German language skills to understand the questionnaires and legal incompetence. Eligible participants completed the questionnaires in the hospital or at home. All participants provided informed consent before completing the questionnaires. The present study was conducted according to the Helsinki declaration and was approved by the local ethics committee (EK026/15).

### Assessment instruments

#### Social attitude item pool

The two item banks assessing social attitude barriers and facilitators towards people with disabilities developed by Garcia and colleagues [4] were translated into German according to the International Society for Pharmacoeconomics and Outcomes Research (ISPOR) translation guidelines [13, 15]. In each item bank, the items were scored on five-point Likert scales ranging from 0 = “never” to 4 = “always”, with higher scores reflecting the perception of more barriers or facilitators, respectively. Three of the authors discussed the content and relevance of the original pool of items (*n* = 82) in the assessment of social attitudes to participation in SpD patients. During this process the item pool was reduced to 53 items by consensus of these authors, including 35 barrier items and 18 facilitator items (see Supplementary Table 1). Thus, items excluded from the German item pool were either items which did not seem to be appropriate for the sample of patients with SpD (e.g., “Because of my disability, criminals see me as an easy target” or “People with disabilities are taken advantage of”) or items with strong content redundancy. To reduce overall response burden, for example, of two items with similar content “People understand my needs for disability accommodations” and “The public respects my needs for disability accommodations”, only the last of the two items was included in the German item pool.

#### Scale validation

Three instruments were used to establish convergent and divergent validity of the Facilitators and Barriers to Social Attitudes item banks, namely the Perceived Social Support Questionnaire (F-SozU-22) [9], the Rasch-based Depression Screening (DESC-I) [16, 17] and the Pain Interference Scale-German (PI-G) [12].

The F-SozU-22 measures five different aspects of perceived social support such as emotional support (F_SozU_E, 8 items), practical support (F_SozU_P, 4 items), social integration (F_SozU_S, 6 items), trustworthiness (F_SozU_V, 2 items) and satisfaction with social support (F_SozU_Z, 2 items). The items have five response options ranging from 1= “does not apply” to 5 = “applies exactly”, with higher scores indicating higher levels of perceived social support. A total score for each subscale was calculated (F_SozU_E = ranging from 5 to 40 points; F_SozU_P = ranging from 5 to 20 points; F_SozU_S = ranging from 5 to 30 points; F_SozU_V = ranging from 5 to 10 points; F_SozU_Z = ranging from 5 to 10 points).

The DESC-I is a ten-item instrument assessing the severity of depressive symptoms during the last 2 weeks, with five response options ranging from 0 = “never” to 4 = “always”. The total score of the scale ranges from 0 to 40 points, and a cut-off ≥12 indicates a possible diagnosis of a depressive episode.

The PI-G assesses the interference of pain on daily functioning during the last 7 days and is composed of three subscales: mental functioning (PIG-Mental, 13 items) to assess cognition and emotion, functional aspect (PIG-FUNC, 11 items), evaluating activities of daily living (ADL), and mobility aspect (PIG-PHYS, 4 items). Response options range from 0 “not at all” to 4 “very much”, from which the total score of each subscale was calculated, with a range from 0 to 52 points for the PIG-Mental, a range from 0 to 44 points for the PIG-FUNC and a range from 0 to 16 points for the PIG-PHYS. In all the PIG subscales, higher value reflects greater pain interference.

### Statistical analysis

The descriptive analysis was performed with the Statistical Package for the Social Sciences (SPSS®) software, version 22.0 (IBM, Armonk, New York, USA). The item analysis according to the Rasch model was carried out using the software RUMM2030.

### Item analysis according to the Rasch model

The data were evaluated using the Rasch model approach [18]. To achieve fit to the model, a series of requirements must be met, including item fit [19], unidimensionality (the items measure a sole latent construct) [7], local independence (the responses to the items are independent given the latent variable) [8] and absence of DIF (the responses of the items and the relevant exogenous variables are independent given the latent variable) [20].

Overall fit to the model was investigated based on the item-trait interaction (*X*^2^-test) [21]. For individual item fit, the standardized residuals were expected to be within a range ± 2.5 [22]. A Bonferroni adjustment was carried out throughout the analyses to avoid unexpected significance (*p* > .01) [23]. For the evaluation of unidimensionality the approach proposed by Smith was used with the criterion that the percentage of significant t-test comparisons should not exceed 5% [22].

A cut-off value of .2 above the average residual correlations (Q3 values) was used to assess LD [8, 24]. There are several possibilities to account for LD. In the framework of item banks, one of the items of the locally dependent pair of items either has to be deleted or deleted and re-entered by using anchoring [25]. In case of scales, LD can either be resolved by deleting one of the locally dependent items or by creating testlets [26].

DIF was assessed for gender and age groups (based on the median value of 56.1 years) using analyses of variance, with the significance level set at 5% and applying a Bonferroni correction. Where appropriate, DIF can be accounted for by splitting the respective item [27].

The person separation index (PSI) determines how well patients can be differentiated. A value >.70 is adequate for group evaluation and > .85 for individual evaluation [26]. Targeting was assessed graphically based on the person-item threshold distribution graph [26, 28]. Additionally, the mean person parameter was compared with the mean item difficulty of the scale.

Additionally, the ordering of the response categories was evaluated by examining the thresholds distribution numerically and graphically [29]. A potential solution for dealing with disordered thresholds is collapsing adjacent categories [30]. Although it should be noted that this is a post-hoc process, and that this does not change the response categories that were initially administered.

### Convergent and divergent validity

Convergent validity was evaluated using Spearman’s rank correlation coefficients. Based on conceptual reasons and findings reported by Wong and colleagues [5], we expected moderate correlations between F-SozU-22, DESC-I, and social attitudes measures, with the highest correlations being expected with the F-SozU-22 subscales, and low correlations between PI-G and social attitudes measures. The correlations between F-SozU-22 and social attitudes scores were expected to be positive for facilitators and negative for barriers [9]. In contrast, negative correlations were expected between PI-G, DESC-I and facilitator scores, and positive correlations between PI-G, DESC-I and barrier scores [12, 16, 17]. In addition, Pearson correlation coefficients were calculated using the interval-scale person parameter to assess the relationship between the facilitator and barrier subscales of the social attitudes, where the facilitator and barrier subscales were expected to be negatively correlated. Correlations between .1 and .3 were considered as weak, between .3 and .5 as medium, and > .5 as strong correlations.

## Results

### Participant’s characteristics

The sample was composed of 246 (60%) women and 163 men (39.8%) (Table 1), with a mean age of 54.4 years (SD ± 15.2; range: 18.8–87.6 years). Three quarters of the participants were outpatients (75.9%), and the most frequent diagnosis was lumbar stenosis (25.6%). Approximately 34% of the sample had a DESC-score above the cut-off for depression (Table 1). They presented moderate levels of impairment related to pain interference, with a mean value of 27.7 (SD: 12.4; range: 0 to 44) for the functional aspect, 24.6 (SD: 11.7; range: 0 to 52) for the mental aspect and 9.0 (SD: 4.7; range: 0 to 16) for the physical aspect.

**Table 1:** Demographic characteristics of the calibration sample

Variable		Total Sample N (%) (*N* = 410) (*N* = 410) (*N* = 410)
Age^a^	< 56.1^b^	204 (49.8)
	≥ 56.1^b^	205 (50.0)
	Missing value	1 (0.2)
Gender	Female	246 (60.0)
	Male	163 (39.8)
	Missing value	1 (0.2)
Type of admittance	Out-patients	311 (75.9)
	In-patients	99 (24.1)
Marital status	Married	236 (57.6)
	Single	65 (15.9)
	Separated/Divorced	50 (12.2)
	Living with partner	25 (6.1)
	Widowed	27 (6.6)
	Others	3 (0.7)
	Declined to respond	4 (1.0)
Current work status	Employed for wages	179 (43.7)
	Retired	101 (24.6)
	disability pension	36 (8.8)
	unemployed	3 (0.7)
	homemaker	33 (8.0)
	vocational training/studies training/studies	5 (1.2)
	partial pension	2 (0.5)
	decline to respond	
Diagnosis	Lumbar stenosis	105 (25.6)
	Lumbar disc herniation	78 (19.0)
	Cervical disc herniation	26 (6.3)
	Cervical myelopathy	28 (6.8)
	Cervical and lumbar	23 (5.6)
	Fracture/trauma	18 (4.4)
	Discitis/spondylodiscitis	16 (3.9)
	Cervical stenosis	14 (3.4)
	Thoracic	11 (2.7)
	Tumor	12 (2.9)
	Ambiguous	63 (15.4)
Patient Reported Outcomes		
Depression	DESC ≥12	140 (34.1)
PI-G subscales		
PIG-Mental	26 (0–46) ^c^	175 (42.7)
PIG-Func	30 (0–44) ^c^	175 (42.7)
PIG-Phys	9 (0–16) ^c^	175 (42.7)
F-SozU-22 subscales		
F_Sozu_S	20 (11–27) ^c^	175 (42.7)
F_Sozu_Z	5 (2–10) ^c^	175 (42.7)
F_Sozu_E	35 (8–40) ^c^	175 (42.7)
F_Sozu_P	18 (4–20) ^c^	175 (42.7)
F_Sozu_V	10 (2–10) ^c^	175 (42.7)

Notes. ^a^ Age range [18.8–87.6 years]; ^b^ median split (median value: 56.1 years old); ^c^ The median and the highest and lowest value of the total score range are reported

### Item analysis according to the Rasch model

An initial assessment of dimensionality of the 18 facilitator items and the 35 barrier items respectively revealed multidimensionality (significant *t-tests:* facilitator items: 24.1%; barrier items: 20.4%). Inspecting the loadings on the first residual factor indicated for both, the facilitator as well as the barrier items, that the items loaded according to two perspectives, individual and group perception (attitudes that individuals with disabilities experienced as being directed towards them vs. attitudes that respondents experienced as being directed towards people with disabilities as a social group). Thus, we departed from Garcia and Wong’s approach of combining individual and societal perceptions, and performed Rasch analyses for four sets of items: 1. Individual facilitators, 2. Societal facilitators, 3. Individual barriers and 4. Societal barriers. This solution resulted in too few items for item bank development. Accordingly, we decided to develop subscales representing four distinct aspects of social attitudes.

All four subscales demonstrated significant misfit to the Rasch model (Table 2). All subscales initially presented misfit of some items and extensive problems related to LD (Table 3), with items B2 (“My family is frustrated with the need to help me because of my disability”) and B3 (“My family acts like my disability is a burden to them”) of the individual barriers subscale having the highest residual correlation (*r* = .56). Moreover, disordered thresholds were observed for many items of the individual facilitators and barriers subscales (Table 3). There was no evidence of gender- or age-related DIF for any of the four subscales.

**Table 2:** Fit statistics for perceived social attitudes measures

Analysis	Item location		Person location		Item-trait interaction (Overall fit to the model)				Reliability (PSI)^c^
	Mean	SD	Mean	SD	***X***^***2***^	df	***p***-value	Unidimensionality^a^	
	**Individual Facilitators**								
Initial	.00	.55	1.17	1.48	122.4	44	.000	%PST = 7.1% (%LB95CI = 4.9%)^b^	.84
Final	.00	.27	.78	1.28	21.8	16	.146	%PST = 2.6%	.69
	**Individual Barriers**								
Initial	.00	.86	−2.99	1.62	601.9	108	.000	%PST = 13.3% (%LB95CI = 10.9%)	.89
Final 14 items	.00	.93	−2.67	1.46	58.7	32	.011*	%PST = 2.6%	.78
Final 7 items	.00	.83	−2.86	1.63	26.1	14	.024*	%PST = 1.6%	.69
	**Societal Facilitators**								
Initial	.00	.41	.52	1.60	84.6	14	.000	%PST = 9.6% (%LB95IC = 7.4%)	.84
Final	.00	.32	.65	2.29	16.3	12	.173	%PST = 3.1%	.87
	**Societal Barriers**								
Initial	.00	.78	−1.37	2.09	99.0	32	.000	%PST = 7.4% (%LB95IC = 5.2%)	.89
Final	.00	.44	−1.07	1.87	18.3	24	.783	%PST = 7.0% (%LB95IC = 4.7%)^b^	.88

Notes. *Good fit of the 14 and 7 items to the model with Bonferroni-adjusted *p* = 0.001; ^a^The percentage of significant t-test comparisons (%PST) should not exceed 5%; ^b^The lower bound of the binomial confidence interval (%LB95IC) below 5% is reported to evidence acceptable unidimensionality. ^c^Person separation index

**Table 3:** Item analysis according to the Rasch model

**1. Individual Facilitators**					
**Item code**	**Item description**	**Initial Analysis: Locally Dependent with Item** **(cut-off value 0.11)**	**Initial Analysis: Item Misfit** **(Standardized Residuals > 2.5 or < − 2.5)**	**Final Solution: Response Categories**^a^	**Final Solution:** **Super items (Testlets of items to account for LD)**
F1	The people in my life accept me for who I am^b^			00012	Testlet A (F1&F3)
F2	The people in my life are sensitive to my disability needs^b^	F3 (*r =* .14); F8 (*r =* .13)			
F3	The people in my life are willing to accommodate my disability^b^	F2 (*r =* .14); F5 (*r =* .11)	−3.796	00012	Testlet A (F1&F3)
F4	People in my life treat me like I can do my own decisions^b^	F5 (*r =* .38); F7 (*r =* .12)	−2.836	00012	Testlet B (F4&F7)
F5	The people in my life treat me with respect^b^	F3 (*r =* .11); F4 (*r =* .38); F7 (*r =* .14)	− 4.295		
F6	The people in my life let me speak for myself^b^	F7 (*r =* .43)			
F7	The people in my life respect that I know best how to take care of myself^b^	F4 (*r =* .12); F5 (*r =* .14); F6 (*r =* .43)		00012	Testlet B (F4&F7)
F8	The public is sensitive to my disability needs	F2 (*r =* .13)	3.712		
F9	People are able to see past my disability	F10 (*r =* .16); F11 (*r =* .12)		01234	
F10	The public respects my needs for disability accommodations^b^	F9 (*r =* .16); F11 (*r =* .21)		00012	Testlet C (F10&F11)
F11	People treat me like a valued member of the community^b^	F9 (*r =* .12); F10 (*r =* .21)		00123	Testlet C (F10&F11)
**2. Societal Facilitators**					
**Item code**	**Item description**	**Initial Analysis: Locally Dependent with Item** **(cut-off value 0.05)**	**Initial Analysis: Item Misfit** **(Standardized Residuals > 2.5 or < −2.5)**	**Final Solution: Response Categories**^a^	**Final Solution:** **Super items (Testlet of items to manage LD)**
F12	People with disabilities are encouraged to participate in my community	F13 (*r =* .28)	6.286		
F13	People with disability are treated fairly at work	F12 (*r =* .28)			
F14	Society is sensitive to the needs of people with disabilities		3.566	01234	Testlet D (F14&F16)
F15	Society is accepting of people with disabilities	F16 (*r =* .23); F18 (*r =* .11)	−2.799	01234	Testlet E (F15&F17&F18)
F16	Society is responsive to the challenge faced by people with disabilities	F15 (*r =* .23); F17 (*r =* .18); F18 (*r =* .26)		01234	Testlet D (F14&F16)
F17	Society values people with disabilities as much as people without disabilities	F16 (*r =* .18); F18 (*r =* .33)		01234	Testlet E (F15&F17&F18)
F18	Society treats people with disabilities fairly	F15 (*r =* .11); F16 (*r =* .26); F17 (*r =* .33)	−2.683	01234	Testlet E (F15&F17&F18)
**3. Individual Barriers**					
**Item code**	**Item description**	**Initial Analysis: Locally Dependent with Item** **(cut-off value 0.17)**	**Initial Analysis: Item Misfit** **(Standardized Residuals > 2.5 or < −2.5)**	**Final Solution: Response Categories**^a^	**Final Solution:** **Super items (Testlet of items to manage LD)**
B1	Because of my disability my family complains that I am to needy^b^	B2 (*r =* .43); B3 (*r =* .36)	4.159		
B2	My family is frustrated with the need to help me because of my disability^b^	B1 (*r =* .43); B3 (*r =* .56)			
B3	My family acts like my disability is a burden to them^b,c^	B1 (*r =* .36); B2 (*r =* .56); B4 (r = .28)		00112	
B4	Because of my disability, my friends spend less time with me^b^	B3 (r = .28)	4.388		
B5	My Friends act like my disability is a burden to them^b^		9.871		
B6	People resent that I get “special treatment” because of my disability		7.539		
B7	Because of my disability, people tell me how to live my life^b^	B8 (*r =* .24)	2.908		
B8	Because of my disability, people avoid me	B7 (*r =* .24); B9 (*r =* .36); B10 (*r =* .28)		01234	Testlet F (B8&B9&B10)
B9	Because of my disability, people exclude me from activities^c^	B8 (*r =* .36)		01234	Testlet F (B8&B9&B10)
B10	Because of my disability, people avoid looking at me	B8 (*r =* .28)		01234	Testlet F (B8&B9&B10)
B11	Because of my disability, people seem uncomfortable with me^c^	B12 (*r =* .30)		01234	
B12	Because of my disability, people are rude to me	B11 (*r =* .30); B15 (*r =* .26); B16 (*r =* .35); B18 (*r =* .20); B23 (*r =* .18); B24 (*r =* .33); B27 (*r =* .19)	−2.757	01234	Testlet I (B12&B15&B16)
B13	People make fun of my disability			01234	Testlet G (B13&B27)
B14	People act as though it is my fault I have this disability				
B15	Because of my disability, people ignore my good qualities^c^	B12 (*r =* .26); B16 (*r =* .38); B18 (*r =* .24); B23 (*r =* .20); B24 (*r =* .22)	−3.401	01234	Testlet I (B12&B15&B16)
B16	Because of my disability, people treat me unfairly	B12 (*r =* .35); B15 (*r =* .38); B24 (*r =* .29); B27 (*r =* .23)	−2.804	01234	Testlet I (B12&B15&B16)
B17	Because of my disability, people stare at me	B18 (*r =* .24); B19 (*r =* .27)			
B18	Because of my disability, people treat me like I’m stupid	B12 (*r =* .20); B15 (*r =* .24); B17 (*r =* .24)	−4.077		
B19	Because of my disability, people treat me like a child^c^	B17 (*r =* .27)	− 3.349		
B20	Because of my disability, people take advantage of me			01234	
B21	Because of my disability, people make decisions for me^c^			01234	Testlet H (B21&B22)
B22	Because of my disability, people speak for me instead of letting me speak for myself			01234	Testlet H (B21&B22)
B23	Because of my disability, people treat me less of a person	B12 (*r =* .18); B15 (*r =* .20);	− 4288		
B24	Because of my disability, people talk down to me	B12 (*r =* .33); B15 (*r =* .22); B16 (*r =* .29)	− 3738		
B25	People are impatient when I take extra time to do things because of my disability^c^			01234	
B26	Because of my disability, people interrupt me when I am talking^b^		− 2578		
B27	People bully me because of my disability	B12 (*r =* .19); B16 (*r =* .23)	− 3451	01234	Testlet G (B13&B27)
**4. Societal Barriers**					
**Item code**	**Item description**	**Initial Analysis: Locally Dependent with Item** **(cut-off value 0.06)**	**Initial Analysis: Item Misfit** **(Standardized Residuals > 2.5 or < − 2.5)**	**Final Solution: Response Categories**^a^	**Final Solution:** **Super items (Testlet of items to manage LD)**
B28	People with disabilities are discriminated against at work		5.253		
B29	Society treats people with disabilities like they are a burden	B30 *(r =* .18)		01234	Testlet K (B29&B30)
B30	Society treats people with disabilities like they are stupid	B29 *(r =* .18)		01234	Testlet K (B29&B30)
B31	Society is unkind to people with disabilities	B30 *(r =* .10); B31 *(r =* .10);		01234	
B32	Society limits the opportunities of people with disabilities	B33 *(r =* .19)		01234	Testlet J (B32&B33&B34)
B33	Society limits the freedom of people with disabilities	B32 *(r =* .19); B34 *(r =* .07)		01234	Testlet J (B32&B33&B34)
B34	Society treats people with disabilities like second-class citizens	B33 *(r =* .07)	−4.101	01234	Testlet J (B32&B33&B34)
B35	Society disrespects people with disabilities			01234	

Notes. Items highlighted in gray correspond to items excluded from the final subscales. In the case of the Individual Barriers subscale, the estimates of the final 14-item long subscale are reported. ^a^Original response categories: 0 = “never”, 1 = “rarely”, 2 = “sometimes”, 3 = “usually”, 4 = “always”. ^b^Initial items with items disordered thresholds. ^c^7 Items of the Individual Barriers subscale short version

We completed several steps to achieve fit to the Rasch model for each subscale: item deletion, rating scale rescoring, and testlet formation to counter LD (Table 3). This approach led to four unidimensional scales with good fit to the Rasch model (Table 2): individual facilitators (7 items; Supplementary Table 2), societal facilitators (5 items; Supplementary Table 3), individual barriers (14 items; Supplementary Table 4) and societal barriers (7 items; Supplementary Table 6). For the individual barriers subscale an additional short scale was developed (7 items) being similar in size as compared to the other three dimensions (Supplementary Table 5).

For the final solutions a raw score-to-measure transformation table is provided, to enable the usage of the interval-level person parameters for further parametric calculations (Supplementary Table 7).

#### Targeting and reliability

**Fig. 1:**
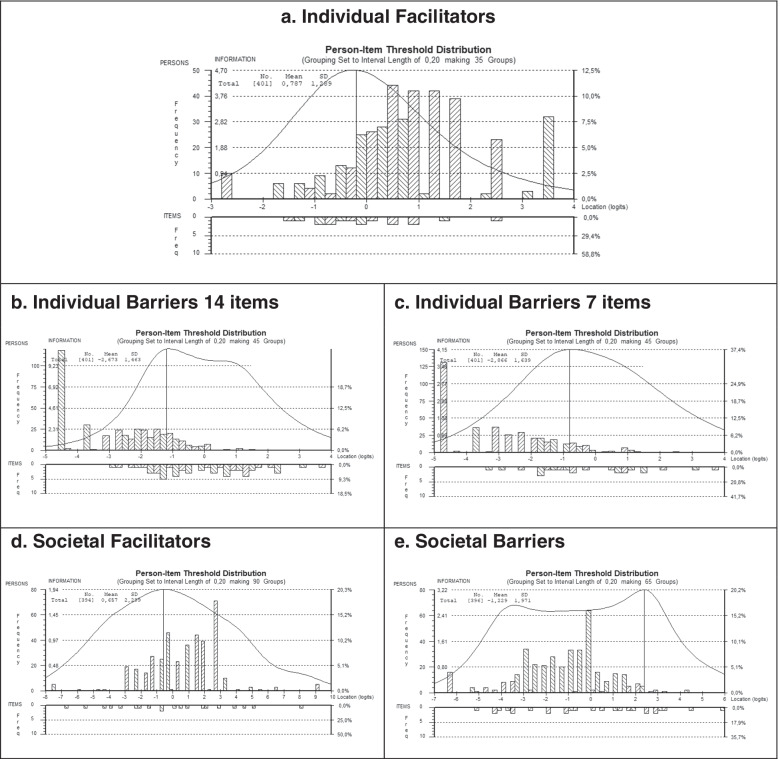
Person-item distribution of the four subscales. Notes. Targeting of the final subscales for in- and out-patients treated for SpD in Germany: **a** Individual Facilitators; **b** and **c**. Individual Barriers; **d**. Societal Facilitators; **e**. Societal Barriers. These person-item distribution graphs show the distribution of participants (top half of graphs) as well as the distribution of item thresholds (bottom half of graphs) across the evaluated dimensions. To achieve a well-targeted scale the average of the persons’ parameters should be close to zero, such as the average of the items’ difficulty. Positive logit values indicate participants with higher levels of the evaluated constructs and more difficult item categories (item thresholds)

Targeting of the four social attitude subscales is presented in Fig. 1. Overall, targeting was good with most patients located in the same range as the item thresholds. However, targeting for this sample was better for subscales assessing group perceptions. Extreme scores were more prevalent for the individual facilitators and barriers with 11.7% at the ceiling for the facilitator scale and 29.9% (IB-14) as well as 35.2% (IB-7) at the floor for the barriers scales. The high number of patients not reporting any individual barriers is also reflected by the low person mean location (IB-14: -2.67; SD =1.46; IB-7: -2.87; SD =1.63; Fig. 1b and c). The better targeting for the two subscales assessing group perceptions also goes along with higher reliability coefficients, with PSI > .85 for both subscales and hence indicating suitability for individual evaluation. Reliability for the individual subscales was .78 for the long version of the individual barriers subscale and .69 for the short version as well as the individual facilitator subscale.

### Convergent and divergent validity

Table 4 shows Pearson’s correlation coefficients between the different versions of the barriers and facilitators subscales and Spearman’s rank correlation coefficients for the evaluation of the correlations between the barriers and facilitators subscales and the F-SozU-22, PI-G and DESC. The long and short version of the individual barriers subscales were highly correlated (*r* = .97). For the individual and societal facilitators subscales, a significant positive correlation of medium size was found (*r =* .45), just as for the individual and social barriers subscales (both IB-subscales: *r =* .34). Individual facilitators and barriers subscales were negatively correlated (IB-14: *r* = −.41; IB-7: *r* = −.39) as were the societal facilitator and barrier subscales (*r* = −.51). The correlations between the social attitudes subscales and the social support-, pain interference- and depression scales were higher for the individual subscales than for the societal subscales, with the correlations for the individual subscales being of medium size.

**Table 4:** Convergent validation

	Social Attitudes measures				
	**IF**	**IB (14 items)**	**IB (7 items)**	**SF**	**SB**
**DESC-I**	−.31**	.31**	.31**	−.20**	.13
	**IF**	**IB (14 items)**	**IB (7 items)**	**SF**	**SB**
**PI-G Subscales**^a^					
**PIG_Mental**	−.34**	.27**	.26**	−.21**	.13
**PIG_FUNC**	−.21**	.23**	.24**	−.21**	.20
**PIG_PHYS**	−.12	.21**	.20**	−.14**	.08
	**IF**	**IB (14 items)**	**IB (7 items)**	**SF**	**SB**
**F-SozU-22 Subscales**^a^					
**F_SozU_E**	.47**	−.32**	−.31**	.25**	−.17*
**F_SozU_P**	.47**	−.30**	−.29**	.25**	−.19**
**F_SozU_S**	.32**	−.24**	−.25**	.26**	−.18*
**F_SozU_V**	.35**	−.25**	−.24**	.16*	−.11
**F_SozU_Z**	−.26**	.23**	.23**	−.09	.16*
	**IF**	**IB (14 items)**	**IB (7 items)**	**SF**	**SB**
**Social Attitudes measures**^b^					
**IF**		−.41**	−.39**	.45**	−.17**
**IB (14 items)**			.97**	−.26**	.34**
**IB (7 items)**				−.25**	.34**
**SF**					−.51**

Notes. ^a^Spearman’s correlations; * *p <* .05; ** *p <* .01. DESC-I: Rasch-based Depression Screening; PI-G: Pain Interference Scale-German; PIG-Mental: Mental functioning; PIG-FUNC: Functional aspect; PIG-PHYS: Mobility aspect; F_SozU_E: Emotional support; F_SozU_P: Practical support; F_SozU_S: Social integration, F_SozU_V: Trustworthiness; F_SozU_Z: Satisfaction with social support. IF: Individual Facilitators; IB (14 items): Individual Barriers – long version; IB (7 items): Individual Barriers – short version; SF: Societal Facilitators; SB: Societal Barriers. ^b^Pearson’s correlation coefficients for the evaluation of correlations between facilitators and barriers subscales of the Social Attitude measures ** *p <* .01

## Discussion

The initial aim of this study was the cultural adaption, calibration and validation of a German version of item banks assessing social attitude facilitators and barriers developed by Garcia and colleagues [4] and Wong and colleagues [5] in a sample of patients treated for SpD. This aim was only partially met. Instead of two item banks, four unidimensional subscales were developed. Unlike for the American version, both the barriers and facilitators item banks had to be divided into two subscales each, in fact into attitudes that individuals with disabilities experience as being directed towards them and attitudes that respondents experience as being directed towards people with disabilities as a social group (societal perception). The resulting four subscales assessing individual perceptions of individual barriers (14 items; short-form: 7 items) and facilitators (7 items) and barriers (7 items) and facilitators (5 items) to participation at the societal level were psychometrically sound after adjusting for LD and the deletion of items due to misfit. None of the four subscales displayed any evidence of DIF with regard to gender or age.

The four subscales demonstrate good targeting to the sample, with better targeting for the two subscales assessing the group perspective. These two subscales also have PSIs >.85, which indicates that they are well suited for individual assessments. In contrast, the subscales assessing individual perspectives presented PSI values between .69 and .74, suggesting their usefulness for group assessments, distinguishing two groups that can be separated with 95% confidence [31]. Moreover, ceiling and floor effects for the subscales assessing individual perspectives contributed to moderate mistargeting. This indicates that this sample with SpD does not perceive pronounced barriers at an individual level (mean person location = − 2.67), but reports more barriers at a societal level (mean person location = − 1.23). The difference between the two perspectives is also reflected by the only medium-sized correlations between the individual and societal level subscales and by the fact that larger correlations between the individual level subscales and the social support, pain interference, and depression scales were found than for those at the societal level.

Our finding that individual and societal perspectives of social barriers and facilitators should be distinguished is in contrast to the results reported by Wong and colleagues [5] who found the individual and societal perception as part of a single dimension. Differences may be the result of the heterogeneous nature of their sample with diverse disabling conditions living in community settings (stroke, traumatic brain injury and spinal cord injury); in contrast, our sample included patients with stenosis or disc herniation who were more independent in activities of daily living and mobility. A comparison between the mean person locations between both studies clearly indicates that the American sample experienced considerably more barriers (mean person locations: Barriers_Wong_ = .94; IB-14 = − 2.67; IB-7 = − 2.86; social barriers = − 1.07; these values are logit scores, and the midpoint of the scales is each anchored at 0 logits – the average item difficulty of each scale). Differences between the two studies might also result from the different approaches to unidimensional analysis. Whereas we applied an independent *t-test* approach, based on the principal component analysis of the residuals (PCA), Wong and colleagues [5] used as the criterion for unidimensionality the unexplained variance in the first principle component analysis.

There were two additional important differences between the two studies. First, in the present study only a few items had to be rescored because of disordered response categories. This difference might originate from the nature of the samples, with our sample being less impaired. Perhaps most importantly, we evaluated and accounted for LD among items whereas Wong and colleagues did not. In our study, LD was a problem in all four subscales and was clearly caused by similar item content. For instance, similarity in content was observed between locally dependent items from the barriers subscales, such as B2 (“My family is frustrated with the need to help me because of my disability”) and B3 (“My family acts like my disability is a burden to them”) (r = .56). Likewise, items with LD from the facilitators subscales also had content redundancy, such as F6 (“The people in my life let me speak for myself”) and F7 (“The people in my life respect that I know best how to take care of myself”) (r = .43). This issue should be investigated carefully in future studies with different diagnosis groups given the large impact that LD might have on parameter and reliability estimation [6, 7, 24].

### Study limitations

The present study revealed an important distinction in social attitudes that was not evaluated by Wong and colleagues [5], social attitudes at the individual vs. societal level. However, the direct comparison between the two studies is only possible to a limited extent, since the differences between the two studies might reflect different inclusion criteria, the different disability groups and a smaller item pool used in the present study. Future studies should focus on the investigation of dimensionality and targeting, as well as LD and DIF across disability groups. A further limitation of the study is that no a-priori sample size calculations were performed. However, given that our scales were well targeted, given that the sample size was big enough to achieve stable parameter estimation [32] and given that we detected problems related to misfit and LD, we are confident that we had sufficient power to identify misfit to the Rasch model.

## Conclusions

The four subscales distinguish an important aspect of social attitudes – individual vs. societal perspectives – that allow accurate and nuanced measurement of social attitudes experienced by people with disabilities.

There are several applications for the social attitudes scales. The subscales of individual barriers and facilitators are well suited for the clinical setting in identifying individual barriers and facilitators that might restrict or support individuals’ participation and health. The subscales of social barriers and facilitators might be more useful at a public policy level to identify problem areas regarding societal attitudes towards people with disability in general and might help to target interventions such as public education strategies.

## Supplementary Information

**Additional file 1: Supplementary Table 1.** Evaluated Item Pool for the Self-report of Social Attitudes toward People with Disabilities.

**Additional file 2: Supplementary Table 2.** Item fit statistics of the individual facilitators subscale sorted by location order in the final analysis.

**Additional file 3: Supplementary Table 3.** Item fit statistics of the societal facilitators subscale sorted by location order in the final analysis.

**Additional file 4: Supplementary Table 4.** Item fit statistics of the individual barriers subscale of 14 items sorted by location order in the final analysis.

**Additional file 5: Supplementary Table 5.** Item fit statistics of the individual barriers subscale of 7 items sorted by location order in the final analysis.

**Additional file 6: Supplementary Table 6.** Item fit statistics of the societal barriers subscale sorted by location order in the final analysis.

**Additional file 7: Supplementary Table 7.** Raw Score to Rasch Parameter Transformation Table.

## Data Availability

The datasets used and/or analyzed during the current study are available from the corresponding author on reasonable request.

## Abbreviations

DIF: Differential Item Functioning
WHO: Word Health Organization
ICF: Disability and Health
SpD: Spinal Diseases
LD: Local Dependence
F-SozU-22: Perceived Social Support Questionnaire
DESC-I: Rasch-based depression screening
PI-G: Pain Interference Scale-German
ICD-10: 10th revision of the International Classification of Diseases
PSI: Person Separation Index
