# Supplementary material for: Development of the German social attitude barriers and facilitators to participation-scales: an analysis according to the Rasch model
Source: BMC Musculoskelet Disord. 2022 May 6;23:423. doi: 10.1186/s12891-022-05339-0 (PMC9074200; doi:10.1186/s12891-022-05339-0)
Supplement: Supplementary file 1 — Additional file 1: Supplementary Table 1. Evaluated Item Pool for the Self-report of Social Attitudes toward People with Disabilities. [file 12891_2022_5339_MOESM1_ESM.pdf]

Supplementary Table 1

Evaluated Item Pool for the Self-report of Social Attitudes toward People with Disabilities

Original response categories: 0= “never”, 1= “rarely”, 2= “sometimes”, 3= “usually”, 4= “always”

Individual Facilitators		
**Item code**	**Item description- Original version**	**Item description- German version**
F1	The people in my life accept me for who I am	Mir nahestehende Menschen akzeptieren mich für das was ich bin.
F2	The people in my life are sensitive to my disability needs	Mir nahestehende Menschen sind sensibel für meine Bedürfnisse, die durch meine Behinderung verursacht sind.
F3	The people in my life are willing to accommodate my disability	Mir nahestehende Menschen sind bereit sich meiner Behinderung anzupassen.
F4	People in my life treat me like I can do my own decisions	Mir nahestehende Menschen lassen mich meine eigenen Entscheidungen treffen.
F5	The people in my life treat me with respect	Mir nahestehende Menschen behandeln mich mit Respekt.
F6	The people in my life let me speak for myself	Mir nahestehende Menschen lassen mich für mich selbst sprechen.
F7	The people in my life respect that I know best how to take care of myself	Mir nahestehende Menschen respektieren, dass ich am besten weiß, wie ich für mich sorgen kann.
F8	The public is sensitive to my disability needs	Andere Menschen sind sensibel für die Bedürfnisse, die durch meine Behinderung verursacht sind.
F9	People are able to see past my disability	Andere Menschen sind in der Lage, über meine Behinderung hinwegzusehen.
F10	The public respects my needs for disability accommodations	Andere Menschen respektieren meinen Bedarf an behindertengerechter Ausstattung und Hilfen.
F11	People treat me like a valued member of the community	Die Leute behandeln mich als vollwertiges Mitglied der Gesellschaft.
**Societal Facilitators**		
**Item code**	**Item description- Original version**	**Item description- German version**
F12	People with disabilities are encouraged to participate in my community	In meiner Umgebung werden Menschen mit Behinderungen zur Teilhabe ermutigt.
F13	People with disability are treated fairly at work	Menschen mit Behinderungen werden bei der Arbeit fair behandelt.
F14	Society is sensitive to the needs of people with disabilities	Die Gesellschaft ist sensibel für die Bedürfnisse von Menschen mit Behinderungen.
F15	Society is accepting of people with disabilities	Die Gesellschaft akzeptiert Menschen mit Behinderung.

F16	Society is responsive to the challenge faced by people with disabilities	Die Gesellschaft geht auf die Probleme, denen Menschen mit Behinderungen gegenüberstehen, ein.
F17	Society values people with disabilities as much as people without disabilities	Die Gesellschaft schätzt Menschen mit Behinderung genauso wie Menschen ohne Behinderung.
F18	Society treats people with disabilities fairly	Die Gesellschaft behandelt Menschen mit Behinderungen fair.
**Individual Barriers**		
**Item code**	**Item description- Original version**	**Item description- German version**
B1	Because of my disability my family complains that I am to needy	Meine Familie beschwert sich, dass ich wegen meiner Behinderung zu hilfsbedürftig sei.
B2	My family is frustrated with the need to help me because of my disability	Meine Familie ist frustriert darüber, mir wegen meiner Behinderung helfen zu müssen.
B3	My family acts like my disability is a burden to them	Meine Familie handelt so, als sei meine Behinderung eine Belastung für sie.
B4	Because of my disability, my friends spend less time with me	Wegen meiner Behinderung verbringen meine Freunde weniger Zeit mit mir.
B5	My Friends act like my disability is a burden to them	Mir nahestehende Menschen handeln so, als ob meine Behinderung eine Belastung für sie sei.
B6	People resent that I get “special treatment” because of my disability	Andere Menschen nehmen mir die „Sonderbehandlung“ wegen meiner Behinderung übel.
B7	Because of my disability, people tell me how to live my life	Aufgrund meiner Behinderung sagen mir die Leute, wie ich mein Leben zu leben habe.
B8	Because of my disability, people avoid me	Wegen meiner Behinderung meiden mich andere Menschen.
B9	Because of my disability, people exclude me from activities	Aufgrund meiner Behinderung schließen mich Leute von Aktivitäten aus.
B10	Because of my disability, people avoid looking at me	Wegen meiner Behinderung vermeiden es die Leute mich anzusehen.
B11	Because of my disability, people seem uncomfortable with me	Wegen meiner Behinderung scheinen Leute sich in meinem Beisein unwohl zu fühlen.
B12	Because of my disability, people are rude to me	Wegen meiner Behinderung sind andere Menschen unfreundlich zu mir.
B13	People make fun of my disability	Die Leute machen sich über meine Behinderung lustig.
B14	People act as though it is my fault I have this disability	Die Leute verhalten sich so, als wäre ich Schuld an meiner Behinderung.
B15	Because of my disability, people ignore my good qualities	Wegen meiner Behinderung ignorieren Menschen meine guten Seiten.
B16	Because of my disability, people treat me unfairly	Wegen meiner Behinderung behandeln mich andere Menschen unfair.
B17	Because of my disability, people stare at me	Wegen meiner Behinderung starren mich andere Menschen an.

B18	Because of my disability, people treat me like I´m stupid	Wegen meiner Behinderung behandeln mich andere Menschen, als ob ich dumm sei.
B19	Because of my disability, people treat me like a child	Wegen meiner Behinderung behandeln mich andere Menschen wie ein Kind.
B20	Because of my disability, people take advantage of me	Wegen meiner Behinderung nutzen mich andere Menschen aus.
B21	Because of my disability, people make decisions for me	Wegen meiner Behinderung treffen andere Menschen Entscheidungen für mich.
B22	Because of my disability, people speak for me instead of letting me speak for myself	Wegen meiner Behinderung sprechen andere Menschen für mich, anstelle mich selbst sprechen zu lassen.
B23	Because of my disability, people treat me less of a person	Wegen meiner Behinderung behandeln mich andere Menschen wie eine minderwertige Person.
B24	Because of my disability, people talk down to me	Wegen meiner Behinderung sprechen andere Menschen herablassend mit mir.
B25	People are impatient when I take extra time to do things because of my disability	Wegen meiner Behinderung sind andere Menschen ungeduldig, wenn ich mehr Zeit benötige, um Dinge zu tun.
B26	Because of my disability, people interrupt me when I am talking	Wegen meiner Behinderung unterbrechen mich andere Menschen, während ich spreche.
B27	People bully me because of my disability	Wegen meiner Behinderung schikanieren mich andere Menschen.
**Societal Barriers**		
**Item code**	**Item description- Original version**	**Item description- German version**
B28	People with disabilities are discriminated against at work	Menschen mit Behinderung werden auf der Arbeit diskriminiert.
B29	Society treats people with disabilities like they are a burden	Die Gesellschaft behandelt Menschen mit Behinderung als seien sie eine Belastung.
B30	Society treats people with disabilities like they are stupid	Die Gesellschaft behandelt Menschen mit Behinderungen als seien sie dumm.
B31	Society is unkind to people with disabilities	Die Gesellschaft ist Menschen mit Behinderungen gegenüber unfreundlich.
B32	Society limits the opportunities of people with disabilities	Die Gesellschaft schränkt die Möglichkeiten von Menschen mit Behinderungen ein.
B33	Society limits the freedom of people with disabilities	Die Gesellschaft schränkt die Freiheit von Menschen mit Behinderungen ein.
B34	Society treats people with disabilities like second-class citizens	Die Gesellschaft behandelt Menschen mit Behinderungen wie Bürger zweiter Klasse.
B35	Society disrespects people with disabilities	Die Gesellschaft respektiert Menschen mit Behinderungen nicht.
