# Supplementary material for: Development of the German social attitude barriers and facilitators to participation-scales: an analysis according to the Rasch model
Source: BMC Musculoskelet Disord. 2022 May 6;23:423. doi: 10.1186/s12891-022-05339-0 (PMC9074200; doi:10.1186/s12891-022-05339-0)
Supplement: Supplementary file 2 — Additional file 2: Supplementary Table 2. Item fit statistics of the individual facilitators subscale sorted by location order in the final analysis. [file 12891_2022_5339_MOESM2_ESM.pdf]

Supplementary Table 2

Item fit statistics of the individual facilitators subscale sorted by location order in the final analysis

	Item	Item Difficulty (logits)	Fit residual (z-values)	χ^2 p-value
Testlet1	F4&F7	-.40	-.24	.13
F4	People in my life treat me like I can do my own decisions			
F7	The people in my life respect that I know best how to take care of myself			
F9	People are able to see past my disability	.05	.77	.62
Testlet2	F1&F3	.15	.16	.19
F1	The people in my life accept me for who I am			
F3	The people in my life are willing to accommodate my disability			
Testlet3	F10&F11	.21	.47	.20
F10	The public respects my needs for disability accommodations			
F11	People treat me like a valuable member of society			
