# Supplementary material for: Development of the German social attitude barriers and facilitators to participation-scales: an analysis according to the Rasch model
Source: BMC Musculoskelet Disord. 2022 May 6;23:423. doi: 10.1186/s12891-022-05339-0 (PMC9074200; doi:10.1186/s12891-022-05339-0)
Supplement: Supplementary file 3 — Additional file 3: Supplementary Table 3. Item fit statistics of the societal facilitators subscale sorted by location order in the final analysis. [file 12891_2022_5339_MOESM3_ESM.pdf]

Supplementary Table 3

Item fit statistics of the societal facilitators subscale sorted by location order in the final analysis.

	Item	Item Difficulty (logits)	Fit residual (z-values)	χ^2 p-value
Testlet1	F14&F16	-.23	-2.12	.05
F14	Society is sensitive to the needs of people with disabilities			
F16	Society is responsive to the challenges faced by people with disabilities			
Testlet2	F15&F17&F18	.23	1.90	.01
F15	Society is accepting of people with disabilities			
F17	Society values people with disabilities as much as people without disabilities			
F18	Society treats people with disabilities fairly			
