# Supplementary material for: Development of the German social attitude barriers and facilitators to participation-scales: an analysis according to the Rasch model
Source: BMC Musculoskelet Disord. 2022 May 6;23:423. doi: 10.1186/s12891-022-05339-0 (PMC9074200; doi:10.1186/s12891-022-05339-0)
Supplement: Supplementary file 4 — Additional file 4: Supplementary Table 4. Item fit statistics of the individual barriers subscale of 14 items sorted by location order in the final analysis. [file 12891_2022_5339_MOESM4_ESM.pdf]

Supplementary Table 4

Item fit statistics of the individual barriers subscale of 14 items sorted by location order in the final analysis

	Item	Item Difficulty (logits)	Fit residual (z-values)	χ^2 p-value
B25	People are impatient when I take extra time to do things because of my disability	-.64	.71	.07
Testlet1	B8&B9&B10	-.48	1.22	.05
B8	Because of my disability, people avoid me			
B9	Because of my disability, people exclude me from activities			
B10	Because of my disability, people avoid looking at me			
B11	Because of my disability, people seem uncomfortable with me	-.17	-.25	.01
Testlet2	B13&B27	.04	-1.26	.09
B13	People make fun of my disability			
B27	People bully me because of my disability			
F20	Because of my disability, people take advantage of me	.09	-.22	.14
Testlet3	B21&B22	.16	1.10	.47
B21	Because of my disability, people make decisions for me			
B22	Because of my disability, people speak for me instead of letting me speak for myself			
Testlet4	B12&B15&B16	.28	-2.09	.33
B12	Because of my disability, people are rude to me			
B15	Because of my disability, people ignore my good qualities			
B16	Because of my disability, people treat me unfairly			
B3	My family acts like my disability is a burden to them	.72	1.48	.56
