# Supplementary material for: Development of the German social attitude barriers and facilitators to participation-scales: an analysis according to the Rasch model
Source: BMC Musculoskelet Disord. 2022 May 6;23:423. doi: 10.1186/s12891-022-05339-0 (PMC9074200; doi:10.1186/s12891-022-05339-0)
Supplement: Supplementary file 5 — Additional file 5: Supplementary Table 5. Item fit statistics of the individual barriers subscale of 7 items sorted by location order in the final analysis. [file 12891_2022_5339_MOESM5_ESM.pdf]

Supplementary Table 5

Item fit statistics of the individual barriers subscale of 7 items sorted by location order in the final analysis

	Item	Item Difficulty (logits)	Fit residual (z-values)	χ^2 p-value
B9	Because of my disability, people exclude me from activities	-.92	1.33	.02
B11	Because of my disability, people seem uncomfortable with me	-.74	-.17	.19
B25	People are impatient when I take extra time to do things because of my disability	-.68	.35	.62
B19	Because of my disability, people treat me like a child	.10	-2.11	.02
B21	Because of my disability, people make decisions for me	.37	.02	.45
B15	Because of my disability, people ignore my good qualities	.46	-1.82	.13
B3	My family acts like my disability is a burden to them	1.41	.49	.07
