# Supplementary material for: Development of the German social attitude barriers and facilitators to participation-scales: an analysis according to the Rasch model
Source: BMC Musculoskelet Disord. 2022 May 6;23:423. doi: 10.1186/s12891-022-05339-0 (PMC9074200; doi:10.1186/s12891-022-05339-0)
Supplement: Supplementary file 6 — Additional file 6: Supplementary Table 6. Item fit statistics of the societal barriers subscale sorted by location order in the final analysis. [file 12891_2022_5339_MOESM6_ESM.pdf]

Supplementary Table 6

Item fit statistics of the societal barriers subscale sorted by location order in the final analysis

	Item	Item Difficulty (logits)	Fit residual (z-values)	χ^2 p-value
Testlet1	B32&B33&B34	-.50	-1.13	.82
B32	Society limits the opportunities of people with disabilities			
B33	Society limits the freedom of people with disabilities			
B34	Society treats people with disabilities like second-class citizens			
Testlet2	B29&B30	-.18	1.04	.88
B29	Society treats people with disabilities like they are a burden			
B30	Society treats people with disabilities like they are stupid			
B35	Society disrespects people with disabilities	-.04	.81	.95
B31	Society is unkind to people with disabilities	.72	.56	.43
