# Supplementary material for: Development of the German social attitude barriers and facilitators to participation-scales: an analysis according to the Rasch model
Source: BMC Musculoskelet Disord. 2022 May 6;23:423. doi: 10.1186/s12891-022-05339-0 (PMC9074200; doi:10.1186/s12891-022-05339-0)
Supplement: Supplementary file 7 — Additional file 7: Supplementary Table 7. Raw Score to Rasch Parameter Transformation Table. [file 12891_2022_5339_MOESM7_ESM.pdf]

Supplementary Table 7

Raw Score to Rasch Parameter Transformation Table

Ordinal scale score	Interval-scaled person estimate									
	IF		IB-14 items		IB-7 items		SF		SB	
	Logits	Re-scaled*	Logits	Re-scaled*	Logits	Re-scaled*	Logits	Re-scaled*	Logits	Re-scaled*
0	-2.674	0	-4.471	0	-4.68	0	-7.481	0	-6.248	0
1	-2.052	10	-3.651	9	-3.773	9	-6.039	9	-5.349	7
2	-1.609	17	-3.127	15	-3.126	16	-4.98	15	-4.719	12
3	-1.295	23	-2.791	18	-2.663	21	-4.202	20	-4.277	15
4	-1.037	27	-2.546	21	-2.295	25	-3.524	24	-3.911	18
5	-0.81	31	-2.353	23	-1.982	28	-2.893	28	-3.571	20
6	-0.599	34	-2.194	25	-1.704	31	-2.302	31	-3.224	23
7	-0.397	37	-2.056	26	-1.448	34	-1.748	35	-2.857	26
8	-0.198	41	-1.934	27	-1.207	36	-1.221	38	-2.469	29
9	0.004	44	-1.823	29	-0.971	39	-0.71	41	-2.071	32
10	0.213	47	-1.719	30	-0.737	41	-0.203	44	-1.674	35
11	0.435	51	-1.622	31	-0.502	43	0.311	47	-1.28	38
12	0.679	55	-1.528	32	-0.262	46	0.843	50	-0.89	41
13	0.958	60	-1.438	33	-0.017	48	1.402	53	-0.501	44
14	1.294	65	-1.349	34	0.235	51	1.997	57	-0.11	47
15	1.733	72	-1.261	35	0.494	54	2.64	61	0.28	50
16	2.401	83	-1.174	36	0.762	56	3.34	65	0.667	53
17	3.415	100	-1.086	37	1.043	59	4.089	70	1.045	56
18			-0.998	37	1.344	63	4.944	75	1.41	58
19			-0.909	38	1.674	66	6.456	84	1.754	61
20			-0.819	39	2.051	70	9.132	100	2.073	63
21			-0.727	40	2.504	75			2.369	66
22			-0.634	41	3.081	81			2.654	68
23			-0.539	42	3.871	89			2.95	70

24			-0.443	43	4.953	100			3.288	73
25			-0.345	45					3.722	76
26			-0.246	46					4.345	81
27			-0.146	47					5.326	88
28			-0.045	48					6.869	100
29			0.057	49						
30			0.16	50						
31			0.263	51						
32			0.365	52						
33			0.468	53						
34			0.57	54						
35			0.672	56						
36			0.773	57						
37			0.874	58						
38			0.976	59						
39			1.078	60						
40			1.183	61						
41			1.29	62						
42			1.402	63						
43			1.52	65						
44			1.646	66						
45			1.783	68						
46			1.932	69						
47			2.098	71						
48			2.284	73						
49			2.497	75						
50			2.747	78						
51			3.05	81						
52			3.438	85						
53			3.995	91						

54			4.792	100						

Notes. This transformation of scores is only valid for complete data. *The Re-scaled value is rounded from 0 to 100 points. **IF:** Individual Facilitators; **IB (14 items):** Individual Barriers – long version; **IB (7 items):** Individual Barriers – short version; **SF:** Societal Facilitators; **SB:** Societal Barriers.
